# Novel Negative Pressure Procedural Tent Reduces Aerosolized Particles in a Simulated Prehospital Setting

**DOI:** 10.1017/S1049023X22000474

**Published:** 2022-06

**Authors:** Nathaniel Hunt, Spencer Masiewicz, Logan Herbert, Benjamin Bassin, Christine Brent, Nathan L. Haas, Mohamad Hakam Tiba, Jon Lillemoen, Mark J. Lowell, Isabel Lott, Matthew Basinger, Graham Smith, Kevin R. Ward

**Affiliations:** 1. Department of Emergency Medicine, University of Michigan, Ann Arbor, Michigan USA; 2. Michigan Center for Integrative Research in Critical Care, Ann Arbor, Michigan USA; 3. Department of Emergency Medicine, Northeast Georgia Medical Center, Gainesville, Georgia USA; 4. Division of Critical Care, Department of Emergency Medicine, University of Michigan, Ann Arbor, Michigan USA; 5. University of Michigan Environment, Health and Safety, Ann Arbor, Michigan USA; 6. University of Michigan Medical School, Ann Arbor, Michigan USA

**Keywords:** aerosol-generating procedures, COVID-19, mitigation, negative pressure tent, prehospital

## Abstract

**Background/Objective:**

The coronavirus disease 2019 (COVID-19) pandemic has challenged the ability of Emergency Medical Services (EMS) providers to maintain personal safety during the treatment and transport of patients potentially infected. Increased rates of COVID-19 infection in EMS providers after patient care exposure, and notably after performing aerosol-generating procedures (AGPs), have been reported. With an already strained workforce seeing rising call volumes and increased risk for AGP-requiring patient presentations, development of novel devices for the protection of EMS providers is of great importance.

Based on the concept of a negative pressure room, the AerosolVE BioDome is designed to encapsulate the patient and contain aerosolized infectious particles produced during AGPs, making the cabin of an EMS vehicle safer for providers. The objective of this study was to determine the efficacy and safety of the tent in mitigating simulated infectious particle spread in varied EMS transport platforms during AGP utilization.

**Methods:**

Fifteen healthy volunteers were enrolled and distributed amongst three EMS vehicles: a ground ambulance, an aeromedical-configured helicopter, and an aeromedical-configured jet. Sodium chloride particles were used to simulate infectious particles and particle counts were obtained in numerous locations close to the tent and around the patient compartment. Counts near the tent were compared to ambient air with and without use of AGPs (non-rebreather mask, continuous positive airway pressure [CPAP] mask, and high-flow nasal cannula [HFNC]).

**Results:**

For all transport platforms, with the tent fan off, the particle generator alone, and with all AGPs produced particle counts inside the tent significantly higher than ambient particle counts (P <.0001). With the tent fan powered on, particle counts near the tent, where EMS providers are expected to be located, showed no significant elevation compared to baseline ambient particle counts during the use of the particle generator alone or with use of any of the AGPs across all transport platforms.

**Conclusion:**

Development of devices to improve safety for EMS providers to allow for use of all available therapies to treat patients while reducing risk of communicable respiratory disease transmission is of paramount importance. The AerosolVE BioDome demonstrated efficacy in creating a negative pressure environment and workspace around the patient and provided significant filtration of simulated respiratory droplets, thus making the confined space of transport vehicles potentially safer for EMS personnel.

## Background

The coronavirus disease 2019 (COVID-19) pandemic has challenged the ability of Emergency Medical Services (EMS) providers to maintain personal safety while providing a range of standard respiratory interventions for patient care at the scene and in transport. Many patients transported by EMS require respiratory therapies, including bag valve mask ventilation, intubation, nebulized medications, continuous positive airway pressure (CPAP) non-invasive ventilation, heated high-flow nasal cannula oxygen therapy (HHFNC), and even surgical airway management. These treatments and procedures are considered aerosol-generating procedures (AGPs) and have the potential to increase spread of infectious viral agents, such as COVID-19, posing an elevated risk of infection to providers utilizing them.^1,2^ Particularly, EMS providers are at risk while performing AGPs given the small, confined space of an ambulance or helicopter and a lack of effective ventilation to mitigate aerosolization of infectious agents. Consequently, many EMS systems have limited the use of AGPs despite the potential patient benefit.

Several studies have found EMS providers can be actively infected during transport of COVID-19 patients. Early in the pandemic, New York City (New York USA) saw a significant increase in 9-1-1 calls for complaints likely to necessitate AGP use, as well as an increase in high-acuity calls, including cardiopulmonary arrest. There was a resultant increased exposure of EMS staff to aerosolizing procedures and risk of contracting COVID-19.^3^ Another study demonstrated that 16.3% of encounters for COVID-19 had one or more EMS-performed AGPs with a subsequent incidence of COVID-19 infection in EMS personnel of 0.57 infections/10,000 person-days.^4^ One study looking specifically at nebulized medication therapy in COVID-19 patients found that 67% of medical personnel developed infection themselves, further noting that the chance of virus transmission would be significantly higher in the limited space of an ambulance compared to in-hospital.^5^ Quarantine after exposure has also been a notable issue with huge implications for the EMS system workforce. A study of 274 EMS encounters with COVID-19 confirmed patients found 151 person-exposures resulting in 981 quarantine days.^6^ The increased necessity for AGPs related to increased volumes of respiratory complaints in the field necessitates updated personal protective equipment (PPE) to minimize prehospital exposures. The risk of COVID-19 infection and/or mandatory quarantine following exposure could potentially be mitigated by novel PPE technologies.

**Figure 1.**
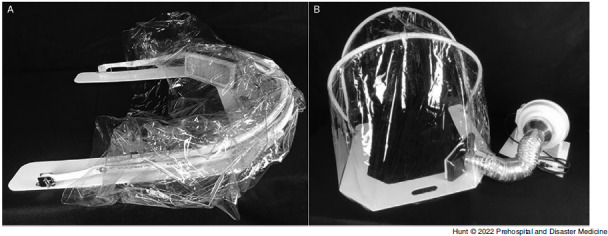
AerosolVE BioDome. Note: A) AerosolVE BioDome in collapsed position; B) AerosolVE BioDome in operating position.

**Figure 2.**
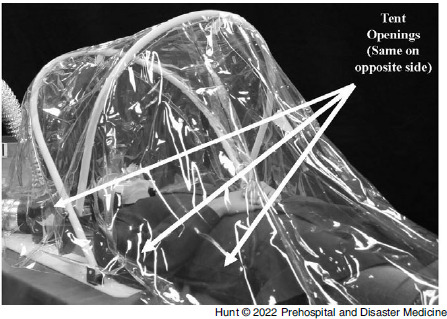
AerosolVE BioDome Access Opening Positions. Note: AerosolVE BioDome with locations of operational openings used during testing. Three identical openings were present on the opposite side.

During the COVID-19 pandemic, the University of Michigan Department of Emergency Medicine (Ann Arbor, Michigan USA), Michigan Center for Integrative Research in Critical Care (M-CIRCC; Ann Arbor, Michigan USA), and the University of Michigan College of Engineering (Ann Arbor, Michigan USA) collaborated with a local manufacturing company to develop a device capable of mitigating these risks. The device (AerosolVE BioDome; Inspire Rx LLC; Ann Arbor, Michigan USA) consists of a clear plastic drape on a collapsible frame that can be secured to a back board or stretcher under the patient. The tent is fitted with a high-efficiency particulate air (HEPA) filter connected to a fan motor. This design allows ambient air to be pulled into the tent and passed through the HEPA filter before being released back into the ambient environment (Figure 1). This creates a negative pressure environment within the tent, allowing infectious particles produced and aerosolized by the patient and/or any AGP to be contained within the tent and filtered before release into ambient air. The device is designed to accommodate use of HHFNC, CPAP/bi-level positive airway pressure (BiPAP), nebulized aerosol therapies, endotracheal intubation/invasive ventilation, and cardiopulmonary resuscitation (CPR). The tent is designed to allow for up to eight entry ports to be cut into the plastic to allow multiple health care workers to access various parts of the patient (Figure 2). The fan motor pulls room air through the tent at 5,600 liters/minute. When compared to current Centers for Disease Control and Prevention (CDC; Atlanta, Georgia USA) recommendations for hospital negative pressure rooms of at least 12 air exchanges per hour (ACH),^7^ the tent produces 900 ACH, or 75-times more air exchanges than current hospital room recommendations. The plastic drape, hose, rigid frame, and backboard are designed to be disposable. The fan motor and filter are designed to be reusable with a filter life of 12 months with continuous use and much longer with intermittent use.

The AerosolVE BioDome and a similar negative pressure helmet were initially developed for use in hospitals. Previous publications have demonstrated their efficacy in reducing air particle counts and in safely allowing advanced AGPs to be performed on COVID-19 patients.^8–11^ The prehospital transport environment varies significantly from a hospital room and transport vehicle platforms (jet versus helicopter versus ground ambulance) are also not equivalent. Thus, testing the device in each different transport platform is integral to ensure efficacy and safety.

The objective of this study was to test the effects of the negative pressure tent device on air particle counts in healthy volunteers undergoing a variety of AGPs in simulated prehospital settings. The hypothesis was the AerosolVE BioDome would prevent increases in air particle counts in the ambient cabin air under a variety of AGPs.

## Methods

This was an open-label study of the efficacy of the AerosolVE BioDome system. The study enrolled 15 healthy volunteers, twelve men and three women, and distributed them amongst three transport platforms: a LearJet 75 configured for medical transport, a Eurocopter EC155 medical helicopter, and a Ford E450 modular ambulance. While not a requirement, all volunteers had been fully vaccinated for COVID-19 prior to participation. Each participant was screened for signs and symptoms of COVID-19 or other respiratory illness prior to enrollment. Sodium chloride particles, generated by a TSI 8026 particle generator (TSI Inc; Shoreview, Minnesota USA), were emitted near the subjects’ mouths to simulate bioaerosol generation associated with viral respiratory infections and to maximally test the system. A TSI 3007 condensation particle counter (TSI Inc; Shoreview, Minnesota USA) was used to detect and quantify air particle counts at different locations, including particle leakage from the tent and particle concentration inside the tent. The device is capable of detecting and quantifying particles in the range of 0.01 to >1.0µm which would include the size of the COVID-19 virus (0.1µm).^12^ This study was approved by the University of Michigan Institutional Review Board and required written consent (protocol # HUM00192223).

**Figure 3.**
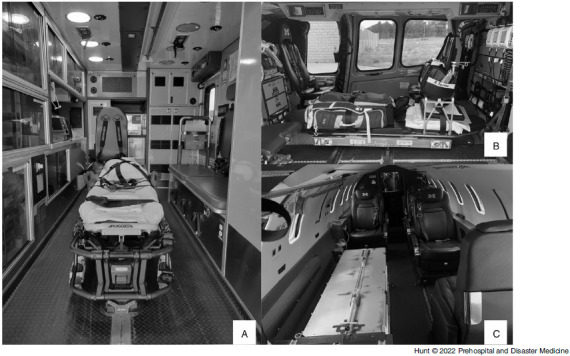
Emergency Medical Services Transport Platforms. Note: Patient compartment view of each transport platform. Driver/pilot compartment not visible. A) Ground Ambulance; B) Medical Helicopter; and C) Medical Jet.

Baseline ambient air particle counts were obtained in the closed cabin of the selected transport platforms without oxygen (O2) devices on and without active particle generation. This determined the ambient particle counts related to dust and other environmental particles. The participant was then placed supine on the tent backboard and the rigid frame and plastic drape placed over them, covering the entire EMS transport stretcher. Six 10cm port holes (two next to the participant’s head, two on each side of the participant) were cut into the plastic to simulate a working configuration necessary for airway management and IV access and management (Figure 2). The particle generator was inserted into the tent through one of the access ports and turned on. This was meant to simulate uniform active expiration and aerosolization of infectious particles from a patient. Particle counts were obtained in numerous locations around the tent and in various locations about the transport platform’s cabin (Figure 3A-C). After baseline counts were obtained without use of an O2 device, the procedure was repeated with the participant wearing a non-rebreather mask at 15L/minute O2 flow, a CPAP mask with pressure of 5cm of water, and a high-flow nasal cannula (HFNC) with 30L/minute of flow. The tests and measurements were performed in succession without any break in the cabin to ensure no inadvertent disruption of ambient particle levels (eg, dust in the outside air entering the cabin that would affect interpretation of subsequent particle counts). The participant was able to remain in the tent for each change of the O2 delivery device. At each testing location, ten particle count measurements were obtained and the mean recorded. This accounted for respiratory variation and other environmental factors that may cause small shifts in particle counts at that location (eg, participants breathing in particles resulting in a momentary reduction in counts inside the tent). Primary outcome was the difference in ambient particle counts and counts close to the tent compared to counts inside the tent with the filter motor off and on. Particle counts in the tent with the motor off were considered a worst-case scenario for AGP production since the boundaries of the tent are in almost identical proximity to where providers will be when caring for a patient undergoing an AGP.

### Statistical Analysis

The primary outcome of particle count inside tent with filter on compared to particle count in the ambient air and the environment close to the tent was analyzed using a one-way ANOVA as this was a comparison of means. Statistical significance was considered as α=0.05. All data were analyzed using PRISM 9 (GraphPad Software; San Diego, California USA).

## Results

Table 1 presents the mean particle counts with and without the use of each O2 delivery device. For all transport platforms, with the tent fan off, the particle generator alone and with all AGPs produced particle counts inside the tent significantly higher than ambient particle counts (P <.0001). With the tent fan powered on, particle counts near the tent, where EMS providers are expected to be located, showed no significant elevation compared to baseline ambient particle counts during the use of the particle generator alone or with use of any of the AGPs. This also held true for other compartments within transport platforms. During the helicopter test series, the ambient particle counts were higher than the other transport platforms, likely due to the environment within the hanger housing the helicopter. Despite this, the fan and filter system were capable of reducing particle counts adjacent to the tent to levels below ambient levels through its entraining of air throughout the helicopter.

**Table 1.** Mean (SD) and 95% CI for Particle Counts for Each Transport Platform and AGP

			Inside Tent		Tent Periphery with Motor On		Ambient Levels with Motor On		
		*Ambient Before Testing Initiated*	Motor Off	Motor On	Near Right Side Abdomen	Filter Exit to Compartment	Jump/Airway Seat	Bench Seat	Driver’s Compartment
**Ambulance**									
Baseline	Mean (SD)	3093 (3667)		4939 (5272)	2161 (2106)	271 (141)	1950 (1792)	1896 (1473)	3863 (2465)
	95%CI (LL)	−1461		−1607	−454	96	-276	67	802
	95%CI (UL)	7647		11485	4777	445	4175	3726	6923
NRB	Mean (SD)	3093 (3667)	25123* (12658)	2727 (3619)	1067 (741)	210 (115)	1239 (1075)	1184 (893)	3444 (2209)
	95%CI (LL)	−1461	9406	−1766	147	68	−96	76	701
	95%CI (UL)	7647	40839	7221	1986	353	2574	2293	6186
CPAP	Mean (SD)	3093 (3667)	35286* (35431)	5674 (5938)	1520 (993)	255 (132)	1523 (1074)	1387 (840)	2932 (1381)
	95%CI (LL)	−1461	−8708	−1699	286	91	190	345	1218
	95%CI (UL)	7647	79279	13047	2753	419	2857	2429	4647
HFNC	Mean (SD)	3093 (3667)	39050* (18747)	5154 (2827)	1927 (1267)	298 (171)	2007 (1605)	1761 (1243)	3724 (1752)
	95%CI (LL)	−1461	15773	1644	353.3	86	14	218	1549
	95%CI (UL)	7647	62327	8664	3500	510	4001	3304	5899
**Helicopter**									
Baseline	Mean (SD)	14130^ (6689)		3101 (1171)	9888 (4189)	1094 (321)	5257 (2373)	5570 (2481)	3958 (1786)
	95%CI (LL)	5825		1647	4686	695	2310	2489	1740
	95%CI (UL)	22436		4555	15090	1493	8204	8651	6175
NRB	Mean (SD)	14130 (6689)	45081* (18691)	6977 (5668)	2231 (506)	414 (76)	1727 (626)	2165 (879)	1790 (805)
	95%CI (LL)	5825	21874	-61	1603	320	950	1073	791
	95%CI (UL)	22436	68289	14015	2859	508	2505	3257	2789
CPAP	Mean (SD)	14130^#^ (6689)	50918* (12546)	12497 (9672)	2198 (1111)	441 (204)	1867 (1056)	2269 (1138)	1717 (847)
	95%CI (LL)	5825	35341	488	818	187	557	856	666
	95%CI (UL)	22436	66496	24506	3578	694	3178	3681	2768
HFNC	Mean (SD)	14130^#^ (6689)	42029* (14834)	9252 (7337)	3173 (1887)	509 (297)	2077 (1335)	2357 (1273)	1753 (912)
	95%CI (LL)	5825	23610	141	831	141	420	777	621
	95%CI (UL)	22436	60448	18363	5516	878	3735	3937	2885
**Jet**									
Baseline	Mean (SD)	840 (677)		1794^&^ (1774)	634.1 (517)	120 (54)	444.4 (309)	422.5 (281)	444 (305)
	95%CI (LL)	0		−410	-8	53	61	74	65
	95%CI (UL)	1680		3997	1276	187	828	771	823
NRB	Mean (SD)	840 (676)	24359* (13767)	3231 (4416)	274.1 (168)	68 (8)	186.3 (79)	189.3 (161)	173 (75)
	95%CI (LL)	0	7265	−252	65	58	88	82	79
	95%CI (UL)	1680	41452	8714	483	79	285	297	266
CPAP	Mean (SD)	840 (677)	47666* (18031)	3703 (3276)	178.1 (94)	67 (10)	163 (67)	160.7 (68)	148 (62)
	95%CI (LL)	0	25277	−365	61	54	80	76	71
	95%CI (UL)	1680	70055	7771	295	80	247	245	225
HFNC	Mean (SD)	840 (677)	29416* (9230)	2420 (1658)	126 (47)	60 (5)	118 (43)	118 (46)	109 (41)
	95%CI (LL)	0	17956	361	68	53	65	62	58
	95%CI (UL)	1680	40877	4479	185	66	172	175	160

Abbreviations: AGP, aerosol-generating procedure; NRB, non-rebreather mask; CPAP, continuous positive airway pressure; HFNC, high-flow nasal cannula.

* Denotes significant difference between “Inside Tent Motor Off” and all other locations (P<.0001).

^ Denotes significant difference between “Ambient Air Level” and “Close to Tent” (P<.0001).

# Denotes significant difference between “Ambient Air Level” and “Filter Exit to Compartment” (P<.0001).

& Denotes significant difference between “Inside Tent” and “Filter Exit to Compartment” (P<.0001).

## Discussion

The results of this analysis demonstrated that with the AerosolVE BioDome activated (fan on), no significant increase in the ambient particle counts near the tent nor in the cabin occurred under all AGP conditions across each transport platform. The particle count in the tent during the AGP without the motor on (levels significantly higher than ambient pre-treatment levels) was used as a surrogate for worst-case particle exposure to an EMS provider close to the patient in the absence of the tent.

The AerosolVE BioDome appears to effectively contain the release of aerosolized particles into the patient care compartment and thus may reduce risks to the patient attendant(s). Interestingly, during the serial tests, a gradual decline in the cabin air particle counts of the various transport vehicles was seen. As the AerosolVE BioDome is designed to entrain cabin air into the tent, which is subsequently filtered, this shows a gradual improvement in air quality and improved safety during the duration of the simulated care event.

For simplicity, the testing protocol excluded the use of a nebulizer, a common respiratory care device used in the prehospital setting, as previous testing had shown similar aerosolized particle generation from the particle generator and the nebulizer.^8^ In seeking to test the device in a “worst-case scenario” (no air exchange), the study did not utilize built-in HVAC (ie, heat, ventilation, air conditioning) in the patient compartment of the ambulance. Any HVAC use would only serve to improve the ambient air quality by creating air exchange.

While a number of negative pressure/isolation “tents” have gained Emergency Use Authorization (EUA) for use within hospitals, none, to the authors’ knowledge, are approved for prehospital use, despite the protracted nature of the COVID-19 pandemic. Furthermore, some of these hospital-use tents rely on regular wall suction to produce a negative pressure within the tent and to prevent AGP-related increases in ambient particles, the efficacy of which has not been reported.

Given the impact on the workforce of infection and/or mandatory quarantine, many EMS systems have reduced their transport of patients with known or highly suspected COVID-19 infections or have modified treatment protocols (eg, using supraglottic airways rather than oral endotracheal intubation, metered-dose inhalers rather than nebulizer treatment) to reduce risks of EMS personnel. The AerosolVE BioDome can likely reduce EMS provider exposure during transport of any patient with unknown COVID-19 status, regardless of symptoms, and allow for maximal patient therapy.

Transport of COVID-19 positive military personnel has seen similar challenges as those described in the civilian EMS sector. Additionally, the necessity for multiple-patient transport is higher in the military sector. Negative pressure transport conexes have been designed to transport multiple infected individuals, but simultaneous use of AGPs within these containment systems has not been reported.^13^ Use of devices like the AerosolVE BioDome may offer additional options and improved safety for care staff.

## Limitations

This study was conducted on healthy volunteers who were breathing normally (not coughing) with simulated infectious droplets dispersed within the tent. The study elected to use a small convenience sample of volunteers rather than a large population as the focus of this study was to determine the device’s efficacy in preventing increases in ambient particle counts in the EMS transport cabin. Given the efficiency of the device in its ability to filter particles, it is unlikely that additional test participants would have made a significant difference in the results obtained. Future studies will be required examining the device and particle counts with real patients being transported and undergoing AGPs. In addition, studies will be required to examine EMS providers’ ability to actively manage and engage patients under the tent for more advanced AGPs such as endotracheal intubation or surgical airway management.

As mentioned above, testing in the helicopter or jet with HVAC cabin air modulation was not possible due to high levels of environmental particles with the aircraft running. While it is believed this creates a “worst-case scenario” in that no cabin air is exchanged, it is difficult to say with certainty how HVAC would have impacted results.

The study design originally intended to use CPAP pressure at 10cm of water and HFNC with 60L/minute of flow. However, these levels of respiratory support were uncomfortable to the healthy volunteers and thus reduced to 5cm of water and 30L/minute, respectively. There were no adverse or safety events reported by participants or observed by the study team during the conduct of this study.

## Conclusion

Given the risk posed by communicable respiratory diseases to prehospital medical providers, novel protective devices may improve safety for these caretakers while still enabling use of respiratory therapies known to increase aerosolization and risk of transmission of infectious agents. Development and testing of these devices are of paramount importance. While this is certainly true during the current pandemic, the same could be said about traditional “respiratory virus season,” when the function of the prehospital system depends heavily on a healthy workforce. The AerosolVE BioDome demonstrated efficacy in creating a negative pressure environment around simulated patients and provided significant filtration of simulated respiratory droplets, thus making the confined space of various EMS transport vehicle types potentially safer for EMS personnel.
